# Similarity of regional brain blood flow and glucose utilization imaging biomarkers in older adults

**DOI:** 10.1002/alz.093808

**Published:** 2025-01-09

**Authors:** Rebecca J Lepping, Jasmine M Taylor, Robyn A Honea, Benjamin J Hess, Eric D Vidoni, William M Brooks, Jeffrey M Burns, Russell H Swerdlow

**Affiliations:** ^1^ Hoglund Biomedical Imaging Center, University of Kansas Medical Center, Kansas City, KS USA; ^2^ University of Kansas Alzheimer’s Disease Research Center, Fairway, KS USA; ^3^ University of Kansas Medical Center, Kansas City, KS USA

## Abstract

**Background:**

Cerebral blood flow (CBF) and glucose utilization have both proven sensitive biomarkers of brain function in Alzheimer’s disease. However, while blood flow supplies glucose to cells to meet local demand, and therefore, are inter‐related, the two aspects are physiologically distinct. Our goal was to conduct a region‐to‐region correlation of magnetic resonance imaging (MRI) and 18F‐fluorodeoxyglucose positron emission tomography (FDG‐PET) biomarkers of cerebral blood flow and glucose utilization to determine whether these physiologically distinct biomarkers yield functionally distinct information.

**Method:**

108 older adults who were cognitively unimpaired (n = 71), or had mild cognitive impairment (n = 17) or dementia due to Alzheimer’s disease (n = 18), and who were enrolled in the KUMC Alzheimer’s Center Cohort completed both MRI T1‐weighted structural and pseudocontinuous arterial spin labelling (PCASL) and FDG‐PET scanning. Participant‐unique brain regions were created by segmentation of the T1‐weighted images with FreeSurfer, combined into large regions of interest for anterior/posterior cingulate, frontal, temporal, and parietal cortices. Preprocessing in SPM12 and AFNI were conducted for the PCASL and FDG‐PET data separately, and regional CBF and glucose utilization (specific uptake value ratio [SUVR]) were extracted. Correlation analysis was conducted in SPSS 29.

**Result:**

CBF and FDG‐PET SUVR were significantly positively correlated in the ACC‐PCC (r = .22, p = .02), Temporal (r = .26, p<.01), and Parietal (r = .37, p<.001) cortices, but not in the Frontal cortex (r = .13, p = .19). The correlations were modest, with the largest at .37 in Parietal cortex.

**Conclusion:**

Our results indicate an expected, yet somewhat modest correlation in the majority compared regions. Additionally, the correlation was not identical across the brain, and non‐significant in frontal regions. This analysis revealed that CBF and FDG‐PET are complementary measures that provide non‐overlapping information. We conclude that these two biomarkers yield unique information and may have unique value in elucidating the relationship between blood flow and neuronal function, potentially more so for frontal rather than parietal regions.

